# Three dimensional three component whole heart cardiovascular magnetic resonance velocity mapping: comparison of flow measurements from 3D and 2D acquisitions

**DOI:** 10.1186/1532-429X-11-3

**Published:** 2009-02-20

**Authors:** Lau Brix, Steffen Ringgaard, Allan Rasmusson, Thomas Sangild Sørensen, W Yong Kim

**Affiliations:** 1 Department of Biomedical Engineering, Region Midtjylland, c/o Aarhus University Hospital, Skejby, Denmark; 2 MR-Centre, Aarhus University Hospital, Skejby, Denmark; 3 Department of Computer Science, University of Aarhus, Aarhus, Denmark; 4 Department of Cardiology, Aarhus University Hospital, Skejby, Denmark; 5 Institute of Clinical Medicine, University of Aarhus, Aarhus, Denmark

## Abstract

**Background:**

Two-dimensional, unidirectionally encoded, cardiovascular magnetic resonance (CMR) velocity mapping is an established technique for the quantification of blood flow in large vessels. However, it requires an operator to correctly align the planes of acquisition. If all three directional components of velocity are measured for each voxel of a 3D volume through the phases of the cardiac cycle, blood flow through any chosen plane can potentially be calculated retrospectively. The initial acquisition is then more time consuming but relatively operator independent.

**Aims:**

To compare the curves and volumes of flow derived from conventional 2D and comprehensive 3D flow acquisitions in a steady state flow model, and in vivo through planes transecting the ascending aorta and pulmonary trunk in 10 healthy volunteers.

**Methods:**

Using a 1.5 T Phillips Intera CMR system, 3D acquisitions used an anisotropic 3D segmented k-space phase contrast gradient echo sequence with a short EPI readout, with prospective ECG and diaphragm navigator gating. The 2D acquisitions used segmented k-space phase contrast with prospective ECG and diaphragm navigator gating. Quantitative flow analyses were performed retrospectively with dedicated software for both the in vivo and in vitro acquisitions.

**Results:**

Analysis of in vitro data found the 3D technique to have overestimated the continuous flow rate by approximately 5% across the entire applied flow range. In vivo, the 2D and the 3D techniques yielded similar volumetric flow curves and measurements. Aortic flow: (mean ± SD), 2D = 89.5 ± 13.5 ml & 3D = 92.7 ± 17.5 ml. Pulmonary flow: 2D = 98.8 ± 18.4 ml & 3D = 94.9 ± 19.0 ml). Each in vivo 3D acquisition took about 8 minutes or more.

**Conclusion:**

Flow measurements derived from the 3D and 2D acquisitions were comparable. Although time consuming, comprehensive 3D velocity acquisition could be relatively operator independent, and could potentially yield information on flow through several retrospectively chosen planes, for example in patients with congenital or valvular heart disease.

## Background

Phase contrast velocity mapping using cardiovascular magnetic resonance (CMR) gives quantitative information of the blood flow in a given vessel and can be performed on a 2D slice or in a 3D volume. The 2D flow measurement is a well established technique for cardiovascular blood flow quantification [1-3]. The approach utilizes 2D slice planes, which must be positioned during scanning requiring a high degree of operator skill. However, by utilizing a 3D phase contrast technique [4,5], the entire heart can be imaged using a 3D volume. Planning of the cardiac scan is hereby reduced to placing a 3D box around the heart, hereby making the acquistion more operator independent. Interactive postprocessing may be used to quantify the blood flow in any arbitrary image plane. This convenient off-line flow evaluation in any imaging plane may overcome the inherent problems related to possible misalignment errors in 2D flow [6].

Besides yielding quantitative information of cardiac global function (*cardiac output, stroke volume, ejection fraction*) the 3D approach (also known as 7D cardiac scan) include qualitative information of the general flow patterns in the cardiac cavities [7], heart valve function (*stenosis*, *regurgitation*) [8] and thoracic aortic flow [9-11]. The visualization of 3D flow patterns by so-called streamlines, arrows or particle paths are based on 3D phase contrast images [12] and have been applied to several regions of the vascular tree [13-15]. Thus, the 3D approach possesses the capacity to provide valuable information that is not available with a 2D technique.

The proposed 3D technique enables automatic slice reformatting in the 3D flow data set, which matches the imaging plane of any arbitrary 2D morphological or functional acquisition. This merging of 3D flow and anatomic images may prove to be a valuable imaging strategy, which can be exploited qualitatively as well as quantitatively. A qualitative aspect is the coloring of the blood flow according to its direction. By doing so, the possibility to differentiate between arteries and veins in the aligned acquisition is enabled. If a morphological scan raises uncertainty regarding the flow direction of a vessel, the 3D flow sequence can provide information otherwise not available. The quantitative aspect possesses the ability to yield accurate flow values through a plane arbitrarily oriented in the acquired 3D volume. Therefore, isotropic 3D whole heart flow measurements may facilitate integration of functional, morphological and quantitative evaluation of complex congenital heart diseases as well as acquired heart diseases [6].

We sought to compare flow curves and volumes derived from conventional 2D and comprehensive 3D CMR phase contrast flow acquisitions in a steady state flow model, and *in vivo *through planes transecting the ascending aorta and pulmonary trunk in healthy volunteers.

## Materials and methods

Ten healthy volunteers (two women and eight men; age 29 ± 7 years) were enrolled in the study. The study was conducted on a whole body 1.5 Tesla Philips Intera CMR system (Philips, Best, The Netherlands) with PowerTrack 6000 gradients. All volunteers underwent the entire CMR examination during a 90–120 minute period, which included subject preparation, scanner set-up and CMR imaging. All data were acquired in the same examination session to ensure comparability.

*In vivo *blood flow measurements were made with a three-dimensional, three-directional phase contrast velocity mapping sequence. The 3D volume was placed in such a way that it covered all of the heart from the apex to the basis including the large thoracic arteries being investigated. Thus, the entire heart was covered with an isotropic spatial resolution of 3.0 × 3.0 × 3.0 mm^3^. All three velocity components were measured by acquiring one segment with no velocity sensitivity and three segments with velocity sensitivity in each direction. Due to the duration of the 3D scans, breath holding was not possible. Instead data acquisition was combined with prospective ECG and diaphragm navigator gating during free breathing with an 8 mm gating window. The 3D flow sequence was a segmented *k*-space phase-contrast gradient echo sequence (4 *k*-lines per heart-phase) with a short EPI readout (EPI factor = 5), hence 20 *k*-lines were acquired per heart phase. TR was 7.6 ms, TE was 4.3 ms, SENSE factor was 1.60 in AP direction and 1.60 in RL direction, flip angle 10°, matrix size 128 × 128, 50 slices and the velocity encoding was set to 150 cm/s. The 3D images were acquired in approximately 8 minutes or more with a navigator efficiency of 50%

The 3D CMR technique was compared with a conventional segmented *k*-space (factor = 3) 2D prospective ECG and diaphragm navigator gated flow sequence with a non-isotropic spatial resolution of 1.41 × 1.41 mm^2 ^and a slice thickness of 5 mm. The flip angle was 25°, TR = 6.4 ms, TE = 3.3 ms, the matrix size was 256 × 256 and 2 signal averages were acquired. The 2D slices were placed approximately 3 cm above the aortic and the pulmonary valves respectively. Both scan techniques used prospective triggering and the navigator was played out for 35 ms at the beginning of each cardiac cycle. The 2D images were acquired in approximately 3 minutes or more with a navigator efficiency of 50%.

The 3D flow measurements were acquired with a temporal resolution of 55 ± 12 ms (20 images per cardiac cycle) while the temporal resolution of the 2D flow measurements was 36 ± 9 ms (29.7 ± 3.7 images per cardiac cycle).

*In vitro *experiments were performed using a closed pumping circuit consisting of a water pump and a silicone tube. The tube was surrounded by stationary water as reference in the isocenter of the magnet. Manganese chloride (0.1 g/L) was added to the steady flowing water to improve the signal intensity at short repetition times. Before each data acquisition the *in vitro *system was manually calibrated with known flow values ranging from 2307 ml/min to 7250 ml/min to simulate realistic blood flow values. This manual flow estimation step was carried out by using a stop watch and a scale. The average of three consecutive manual flow measurements was then used as the true flow value when compared to the CMR obtained flow values. The imaging parameters of the acquired images were identical to the *in vivo *experiment. Immediately after each calibration, sets of paired observations were obtained using the 2D and 3D CMR methods respectively.

### Data Evaluation

**Figure 1:**
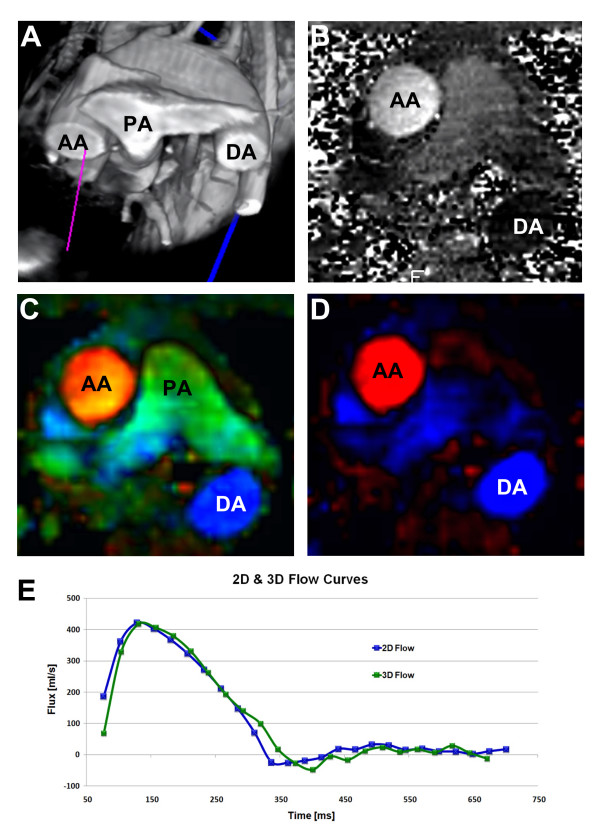
**(A), a volume rendered morphological 3D view**. (B) 2D phase contrast image of the aortic blood. (C) Alignment of the 2D slice in (B) into the 3D flow volume. In-plane flow of the 3D flow acquisition is described by a green color component. (D) Same slice as in (C) showing the through-plane components of the 3D flow acquisition in blue/red. (E) Flow curves from the 2D flow scan in (B) and the aligned slice from the 3D flow scan in (D). Abbreviations: AA: Ascending Aorta. DA: Descending Aorta. PA: Pulmonary Artery.

All data analyses were done by dedicated computer software developed for this study. Based on information of position and orientation from the acquisitions, the software automatically aligned the 2D flow slices into the 3D flow volume. The position in the 3D data set was visually compared with the 2D slice before analyses were performed. If the position in the 3D data set was not perfectly matched, the software allowed the user to manually change the position in the 3D flow data set. To ease this step a 3D morphology scan was used to visually guide the user to match the 2D and 3D flow analysis (Fig. 1). Regions of interest (ROI) could then be placed on the ascending aorta and the pulmonary arteries and the flow values calculated.

Data were evaluated for normality by quantile-quantile plots and statistical comparisons of the 2D and 3D methods were done by paired t-tests and by Bland & Altman plots. All statistical analyses were performed in Microsoft Excel 2003 with the statistical add-in tool pack Analyse-It^® ^Software, Ltd (Version 1.73). All results are presented as mean ± standard deviation. P values ≤ 0.05 were considered statistically significant.

## Results

**Figure 2:**
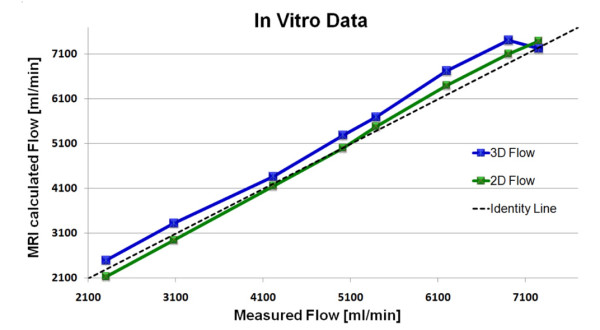
**Graph showing the paired flow *in vitro *values using the 2D and 3D techniques**. The dotted line represents the true flow.

The 2D *in vitro *flow measurements were not different from the true flow measured by bucket and stopwatch (p = 0.30). However, the 3D flow estimations were significantly higher than the true flow (p = 0.004) with a mean difference of approximately 5% (275 ± 168 ml/min). The overestimation of the 3D flow measurements was present in the entire flow range as indicated by Figure 2. A significant difference was also found between the 2D and 3D flow techniques (p = 0.020). The 3D flow was 4% higher than the 2D flow (mean difference 214 ± 189 ml/min).

Seventeen complete data sets were successfully obtained from the volunteers in the *in vivo *experiments. Aortic flow data from one volunteer was rejected due to a faulty 2D slice placement directly on top of the aortic valve. A 3D flow data set was rejected from one subject due to an ECG-triggering fault, which made 3D data acquisition impossible. All measured flow data are presented in Table 1.

**Table 1:** Results from the 2D and 3D in vivo flow data

Volunteer #	2D Aortic Flow [ml/stroke]	3D Aortic Flow [ml/stroke]	2D Pulmonary Flow [ml/stroke]	3D Pulmonary Flow [ml/stroke]
1	81.93	75.92	81.17	80.71
2	91.42	84.39	89.94	77.34
3	81.56	65.68	70.04	60.66
4	76.64	86.77	70.17	104.23
5	118.66	112.72	104.39	107.24
6	111.03	114.87	90.82	115.24
7	124.27	111.13	104.24	91.01
8	105.1	107.28	103.68	106.28
9	113.5^a^	82.49	90.77	91.59
10	95.48	-^b^	90.56	-^b^

^a ^Data rejected due to a faulty 2D slice placement directly on top of the aortic valve

^b ^Data rejected due to ECG-triggering failure during data acquisition

The 3D and the 2D flow images were acquired in 503.7 ± 39.3 seconds and 162.6 ± 54.8 seconds respectively. Results from the aortic flow measurements yielded 2D stroke values of 89.5 ± 13.5 ml/stroke and 3D stroke values of 92.7 ± 17.5 ml/stroke (p = 0.68, mean difference 4.0 ± 8.8 ml). The pulmonary flow data resulted in a 2D stroke volume of 98.8 ± 18.4 ml/stroke and a 3D stroke volume of 94.9 ± 19.0 ml/stroke (p = 0.67, mean difference 3.2 ± 16.2 ml).

**Figure 3:**
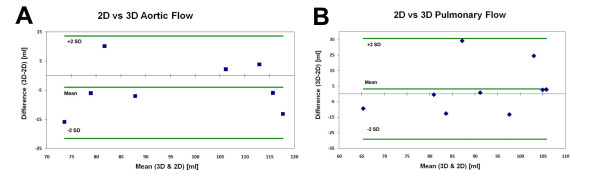
**Bland & Altman Plots showing the differences in flow data between the 2D & 3D methods**. (A), Bland & Altman Plot of the aortic flow. (B), Bland & Altman Plot of the pulmonary flow.

The results show no statistical differences between the 3D and 2D *in vivo *flow measurements. Bland & Altman plots of the data did not suggest that a systematic difference was present in the acquired data as indicated by the data point distribution (Fig. 3).

## Discussion

### *In Vitro *Study

The applied 2D phase-contrast sequence follows the protocol prescription described by Bakker *et al *[16]. The robustness of the 2D technique is supported by the findings of Chatzimavroudis *et al *[17] as well as our *in vitro *data. Our results from the *in vitro *study suggest that the presented 3D technique overestimates the true steady state flow by approximately 5%. This overestimation seems to apply along the entire range of the applied flow values as indicated by Figure 2. Several systematic errors may have contributed to this including eddy currents, Maxwell terms error, partial volume effects and gradient non-linearities.

However, the data reported in our *in vitro *study does not necessarily apply to the *in vivo *situation for several reasons. The water used in our study is an imperfect substitute for human blood and the flow system utilized laminar steady state flow rather than pulsatile. Thus, a direct comparison of our *in vitro *and *in vivo *data may be somewhat misleading and care must be taken when evaluating the precision of the applied 3D flow protocol *in vivo*.

In a clinical setting an error of up to 10% can be expected using 2D phase contrast measurements [1]. We found an overestimation with the 3D protocol of approximately 5% equally distributed over the applied flow range. We therefore argue that this increase is acceptable and the 3D technique is a feasible approach for operator-independent flow imaging.

### *In Vivo *Study

Our *in vivo *results suggest that the two blood flow measurement methods provide similar flow results. Qualitative 3D flow techniques have been implemented in a variety of clinical areas including the great vessels, the thoracic aorta, the left ventricle and the outflow tract, the left atrium, and the aortic valve. Several studies have validated 3D flow against the conventional 2D approach [18,19]. The feasibility of using isotropic 3D CMR data has already been shown on morphological data [20]. However, this study aims to enhance this approach to include flow data. By using isotropic voxels, the 3D technique may be truly operator independent and all imaging planes are acquired during a single acquisition as shown in this study. Our 3D approach had a lower spatial and temporal resolution as compared to previous studies [18,19]. In the future, this may be overcome by improvements in hardware and software, e.g. using parallel imaging and under sampling methods such as *k-t *SENSE [21-23]. Due to the use of navigator corrected respiratory motion compensation, approximately 77 ms of the initial cardiac cycle was not measured. Therefore we had to interpolate the first time point in the flow curve.

### Scan time

The scan time of the 3D technique is rather long compared to the applied 2D protocol. This is in concordance with previous studies [18,19]. However, in order to accurately quantify blood flow, the 2D slice must be placed perpendicular to the vessel. To do this, one or more reference images must be taken to ensure that the slice is correctly angulated. Thus, to obtain a 2D flow measurement of the pulmonary artery and the aorta, a minimum of four scans must be acquired. By using 3D acquisition with isotropic voxels, a single 3D volume can be placed around the entire heart, obviating the need for reference scans and therefore the total examination time will only be slightly increased as compared to the traditional method.

### Spatial resolution

One disadvantage of the 3D method is the lower spatial resolution as compared with the traditional 2D approach. This limitation is dictated by the scan time limitation. The lower spatial resolution will, of course, render more blurry images than for 2D, but for accurate flow assessment in larger vessel high spatial resolution is not critical. As shown by Hofman *et al *[24], the volume flow can be accurately determined as long as there are more than 3 pixels across the vessel. Thus, with a 3 mm isotropic resolution, the flow can be accurately determined in vessels with a diameter larger than 9 mm.

### Study limitations

The use of a single velocity encoding range for all regions of the 3D volume may be suboptimal if regions of high velocity jet flow needed to be measured as well as low velocity venous or arterial flows. The use of prospective ECG gating fails to provide information on late diastolic (atrial systolic) flow. Furthermore, there was slight loss of data on early systolic arterial flow due to the pre-systolic placement of the navigator pulse. Late diastolic navigator placement may therefore be preferable in most cases.

Finally, until robust and user-friendly software becomes available commercially, expert and time consuming post processing of 3D data is needed.

## Conclusion

Three dimensional, three directionally encoded velocity mapping offers a relatively comprehensive and operator independent but more time consuming method for deriving curves and volumes of flow through retrospectively chosen planes of measurement. Aortic and pulmonary flow measurements compared well with those from conventional 2D, through-plane velocity acquisitions in the volunteers studied. Subject to the limitations outlined, the comprehensive 3D approach has the potential to be of clinical value in patients with congenital or valvular heart disease, for example in the presence of shunts or other cardiovascular malformations.

## Competing interests

The authors declare that they have no competing interests.

## Authors' contributions

LB carried out all the CMR scans, set up the applied protocols, analyzed the acquired CMR data, did the statistical analyses, obtained all illustrations and tables, wrote the manuscript and merged all feedback from the co-authors into the final manuscript. SR contributed to the sequence setup, revised the manuscript critically and contributed to important intellectual content of the manuscript. AR contributed to software development. TSS contributed to software development, the study design, drafting the manuscript and revised it critically for intellectual content. WYK contributed to the study design, drafting the manuscript and revised it critically for intellectual content. All authors have read and approved the final manuscript.
